# Retracted: Influence of Two Common Polymorphisms in the *EPHX1* Gene on Warfarin Maintenance Dosage: A Meta-Analysis

**DOI:** 10.1155/2019/6910869

**Published:** 2019-03-07

**Authors:** BioMed Research International

BioMed Research International has retracted the article titled “Influence of Two Common Polymorphisms in the* EPHX1* Gene on Warfarin Maintenance Dosage: A Meta-Analysis” [1]. This article is one of a series of very similar meta-analyses written by different authors which were published in 2014 and 2015, characterized by searching the complementary and alternative medicine database CISCOM despite the topic not being about CAM [2]. The overlaps of language expression with these articles are concentrated in the Materials and Methods, Results, and Discussion sections. In the article, the restriction to two EPHX1 polymorphisms was not justified and the change of one of the SNPs from rs2292566 to rs1131873 in 2013 was not mentioned.

The authors clarified that, a few years ago, they took a training course on how to write meta-analyses, in China, where they were provided with some templates. They said that they referred to these templates to write the article and sought help from a native English speaker. In addition, the corresponding author was not aware of the existence of the article before its publication. They confirmed the concerns above and asked to retract the article due to it being flawed.
